# Spatiotemporal Dynamics and Co-Occurrence Patterns of Marine Fungal Communities Along Nutrient Gradients in the Leizhou Peninsula, China

**DOI:** 10.3390/jof12040260

**Published:** 2026-04-03

**Authors:** Yingyi Fan, Menghan Gao, Bihong Liu, Junyu Wei, Jianming Li, Zhangxi Hu

**Affiliations:** Guangdong Provincial Key Laboratory of Aquatic Animal Disease Control and Healthy Culture, Laboratory of Marine Ecology and Aquaculture Environment of Zhanjiang, College of Fisheries, Guangdong Ocean University, Zhanjiang 524088, China; fanyingyi1@stu.gdou.edu.cn (Y.F.); gaomenghan0914@163.com (M.G.); 2112101021@stu.gdou.edu.cn (B.L.); arctichakimifish@gmail.com (J.W.); 13434643014@stu.gdou.edu.cn (J.L.)

**Keywords:** marine fungi, Leizhou Peninsula, dissolved inorganic nitrogen, spatiotemporal dynamics, co-occurrence network

## Abstract

Marine fungi are pivotal components of coastal ecosystems, facilitating essential biogeochemical cycling and trophic dynamics. However, the complex mechanisms governing their spatiotemporal community patterns in tropical–subtropical coasts remain largely unexplored. In this study, we characterized marine fungal diversity across a comprehensive seasonal cycle (spring (March), summer (June), autumn (August), and winter (December)) at 21 representative sites along the Leizhou Peninsula, China. These sites were strategically selected to encompass a broad range of dissolved inorganic nitrogen (DIN) gradients. Fungal community composition was characterized via high-throughput sequencing of the internal transcribed spacer 2 (ITS2) region, followed by functional guild profiling using the FUNGuild database. A total of 8777 amplicon sequence variants (ASVs) were identified, encompassing a broad taxonomic breadth of 10 phyla and 358 genera. Ascomycota, Basidiomycota, and Chytridiomycota emerged as the predominant phyla across all samples. Our results revealed significant spatiotemporal heterogeneities: seasonal succession fundamentally reshaped community composition, with DIN exerting its most pronounced influence during the winter. Furthermore, fungal functional structures exhibited distinctive clustering across regions defined by DIN enrichment levels. Co-occurrence network analysis revealed a highly modular and robust architecture, characterized by predominantly positive interactions and dense inter-taxon connectivity. These findings underscore the synergistic influence of temporal dynamics and DIN enrichment in shaping marine fungal community assembly and functional compositions. Our study provides critical baseline insights into the ecological resilience of coastal mycobiota in the South China Sea.

## 1. Introduction

Currently, “marine fungi” are defined as species that are not only frequently isolated from marine habitats but also functionally adapted to them, including the capacity to vegetatively grow, complete reproductive cycles, establish symbioses, and maintain metabolic plasticity in marine conditions [1]. Marine fungi are multifaceted ecological drivers acting as essential saprotrophs, parasites, pathogens, as well as forming complex symbiotic and mutualistic associations [2]. Consequently, a comprehensive understanding of the distributional patterns and community structure of marine fungi is fundamental to deciphering their multifaceted ecological roles in coastal environments. Planktonic fungi play key biogeochemical roles in coastal ecosystems, primarily regulating carbon (C), nitrogen (N), and phosphorus (P) cycling through extracellular enzyme–mediated saprotrophic activity [1]. Within the marine carbon cycle, fungi associated with particulate organic matter (POM) degrade complex organic substrates into dissolved organic matter (DOM), contributing to C remineralization [3,4].

Beyond their roles in C sequestration and decomposition, marine fungi mediate N cycling and associated energy transduction pathways. Aquatic fungi are capable of acquiring N through the decomposition of organic matter, even in N-limited environments [5,6,7,8,9]. Under elevated dissolved inorganic nitrogen (DIN) and eutrophic conditions, fungi may contribute to N transformation via denitrification and related processes, potentially affecting nitrous oxide (N_2_O) production and emissions. Nevertheless, how fungal communities in coastal waters spatially respond to DIN gradients remains poorly understood and lacks systematic investigation.

Marine fungi not only regulate biogeochemical cycling through the decomposition of POM, but also influence phytoplankton community dynamics via parasitic interactions. Saprotrophic particle-associated fungi dominated by Ascomycota and Basidiomycota can process algal-derived polysaccharides and POM [10,11,12,13]. In contrast, parasitic fungi, such as Chytridiomycota (“chytrids”) [14,15,16,17], infect marine phytoplankton and influence their population dynamics and organic matter transfer among different phytoplankton taxa. Through these processes, fungal parasitism indirectly influences primary production and the transfer of C to higher trophic levels. Such fungal-mediated regulation of phytoplankton communities and organic matter pathways may be particularly important in coastal systems experiencing increased nutrient loading [18,19,20,21].

In coastal waters, eutrophication is typically caused by excessive nutrient inputs and imbalanced ratios of N and P, which alter nutrient availability and subsequently influence the flow of organic matter within food webs [22]. Since the Industrial Revolution, intensified anthropogenic activities have increased nutrient inputs and caused widespread eutrophication in China’s coastal waters [23,24]. In nearshore systems, eutrophication is primarily driven by N loading, and DIN is commonly used as a key indicator of eutrophication intensity. Elevated nutrient levels can trigger harmful algal blooms (HABs) and ultimately lead to the degradation of marine ecosystem functions [25].

As key decomposers, marine fungi participate in the biogeochemical cycling of essential nutrients, and eutrophication intensity strongly influences fungal community composition and assembly. Previous studies have demonstrated that eutrophication significantly alters planktonic fungal communities in the Beibu Gulf, where rare fungal taxa are more sensitive to changes in N compounds, suggesting a potentially active role of rare planktonic fungi in N utilization in this region [26]. Moreover, eutrophication may increase the abundance of opportunistic or potentially pathogenic fungi, thereby reshaping the functional composition of marine fungal communities [27]. Given the differential responses of marine fungi to various N forms, spatial partitioning based on DIN gradients provides a useful framework for understanding the assembly of marine fungal communities. Furthermore, marine fungi produce diverse bioactive compounds [28,29] and some species can suppress harmful bloom-forming microalgae through secondary metabolites, suggesting that marine fungi are not only responders to eutrophication-driven environmental change but also active participants in material cycling and community regulation in coastal ecosystems [30].

Marine fungi have long been overlooked in marine ecosystems, once regarded as a “fungal desert” [18]. However, with the increasing number of studies on marine fungi, scientific understanding of this group has expanded substantially. Advances in molecular biological techniques have further revealed the high diversity of marine fungi and highlighted their roles in biogeochemical cycling and ecological processes.

As of November 2025, a total of 2253 marine fungal species have been formally described (www.marinefungi.org, accessed on 23 November 2025). However, multiple studies suggest that the currently described species may represent only approximately 1% of the actual marine fungal diversity [31,32,33]. Consequently, a large proportion of marine fungal species remain undescribed, and their associated ecological functions have not yet been adequately resolved [34,35]. As a result, the diversity and distribution patterns of marine fungal communities remain insufficiently studied. Currently, research on the diversity, abundance, and ecological roles of planktonic fungi is largely limited to specific geographic regions [18]. In the Zhanjiang region of China, most studies have focused on mangrove intertidal zones and endophytic fungi within mangroves, while most relevant investigations have mainly examined intertidal zones, sediments, and adjacent coastal waters [36,37,38,39,40]. In contrast, systematic surveys of planktonic fungal communities in the water column of nearby offshore areas remain scarce, especially regarding their spatial distribution and environmental drivers. Debeljak, Baltar and others have reported that fungal diversity tends to be highest nearshore and gradually decreases toward offshore areas, suggesting that the “land–sea connectivity” is a key determinant of this pattern [41,42,43]. Similarly, Clipson et al. noted that “Near-shore marine ecosystems are not as stable as their open ocean counterparts and can vary in input of terrestrial organic matter, sediment, nutrients, and pollutants” [44,45]. This environmental heterogeneity is likely to exert significant influence on microbial community structure and distribution.

Leizhou Peninsula is located at the southernmost tip of the Chinese mainland, surrounded by the sea on three sides, with the South China Sea to the east, the Beibu Gulf to the west, and the Qiongzhou Strait to the south. Its adjacent waters in the northern South China Sea are influenced by multiple hydrological factors, including the subtropical monsoon, the South China Sea Warm Current, and riverine discharge. The coastal waters of the Leizhou Peninsula represent a convergence zone between continental and marine systems. In addition, numerous aquaculture areas and ports mean that industrial, agricultural, and domestic wastewater significantly influence water quality [46]. Against this background, we employed high-resolution ITS2 amplicon sequencing [47] to characterize fungal communities in water samples collected from 21 coastal sites along the Leizhou Peninsula (Zhanjiang, China), in conjunction with measurements of key environmental variables. This study systematically investigates the diversity, temporal dynamics, and spatial distribution patterns of marine fungal communities along DIN gradients. By analyzing the relationships between fungal community structure and site-specific environmental factors, we aim to establish baseline data for understanding how coastal fungal communities respond to both natural environmental fluctuations and anthropogenic impacts, thereby addressing critical knowledge gaps regarding marine fungal diversity in the coastal waters of the Leizhou Peninsula.

## 2. Materials and Methods

### 2.1. Sampling Sites and Sample Collection

**Figure 1:**
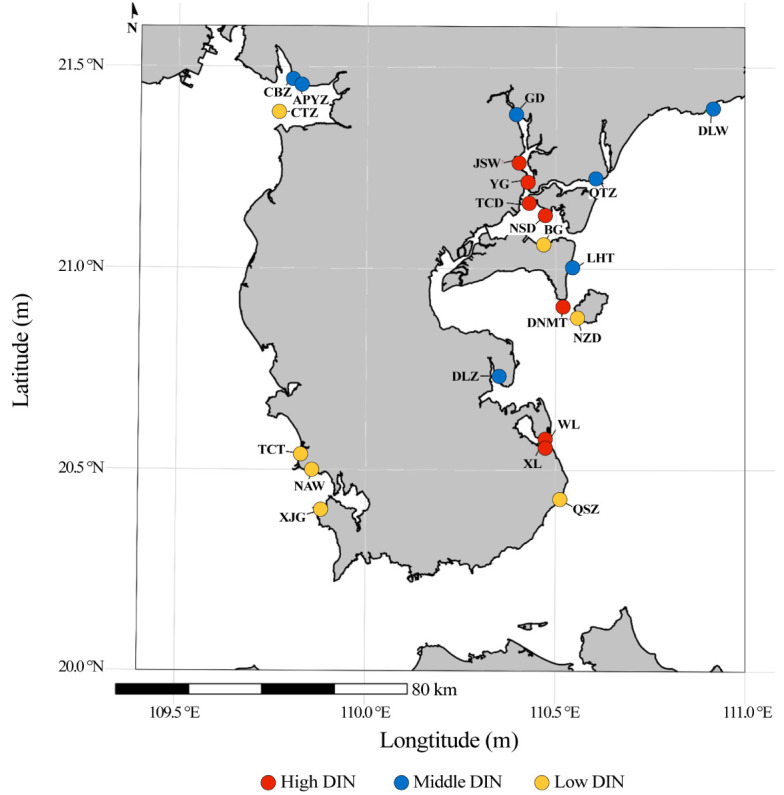
Sampling sites in the coastal waters of the Leizhou Peninsula, China. Letters in the figure denote abbreviations of the site names.

Surface seawater was sampled seasonally from August 2022 to June 2023 to capture four seasons: autumn (August 2022), winter (December 2022), spring (March 2023), and summer (June 2023). Twenty-one sampling stations were distributed within the coastal waters of the Leizhou Peninsula, China (Figure 1; Table S1). Seawater samples were collected in triplicate (5 L per replicate) from the surface layer (0–1.0 m) at each station. Upon collection, samples were immediately chilled and maintained below 4 °C in the laboratory. To collect planktonic fungal biomass, the water samples were filtered through 0.2 µm pore-size membranes (Pall Corporation, Ann Arbor, MI, USA). Following filtration, the membranes were aseptically transferred into sterile cryovials and stored at −80 °C for subsequent metabarcoding analyses.

### 2.2. Environmental Factor Analyses

At each sampling station, surface water temperature, salinity, pH, and in vivo chlorophyll *a* were obtained by a portable multi-parameter water quality analyzer (LH-T600, Zhejiang Lohand Environment Technology Co., Ltd., Hangzhou, China). Water depth was measured using a depth sounder (SM-5A, Speedtech, Shanghai, China). Seawater samples for nutrients and chlorophyll *a* analyses were filtered through 0.45 µm cellulose acetate membranes, whereas samples for chemical oxygen demand (COD) determination were immediately refrigerated after collection. Concentrations of NO_3_^−^-N, NO_2_^−^-N, NH_4_^+^-N, PO_4_^3−^-P, and SiO_3_^2−^-Si were determined using an automatic nutrient analyzer (Smart 200, Alliance, France). DIN was calculated as the sum of NO_3_^−^-N, NO_2_^−^-N, and NH_4_^+^-N. COD was measured using the alkaline potassium permanganate method. All laboratory analyses were completed within 48 h of sample collection.

### 2.3. DNA Extraction, PCR Amplification, ITS Sequencing, Data Processing, and Bioinformatic Analyses

Total genomic DNA was extracted using the CTAB method with nuclear-free water for blank according to the manufacturer’s instructions. The 5′ ends of the primers were tagged with specific barcodes per sample and sequencing universal primers. The ITS2 region [48] was amplified using the primers fITS7 (5′-GTGARTCATCGAATCTTTG-3′) and ITS4 (5′-TCCTCCGCTTATTGATATGC-3′). The PCR conditions to amplify the ITS fragments consisted of an initial denaturation at 98 °C for 30 s, 32 cycles of denaturation at 98 °C for 10 s, annealing at 54 °C for 30 s, and extension at 72 °C for 45 s; and then final extension at 72 °C for 10 min. PCR amplification was performed in a 25 μL reaction mixture with 25 ng template DNA, 12.5 μL PCR Premix, 2.5 μL of each primer, and PCR-grade water. The PCR products were confirmed with 2% agarose gel electrophoresis. Ultrapure water was used as a negative control to prevent false-positive PCR results. The PCR products were purified by AMPure XT beads (Beckman Coulter Genomics, Danvers, MA, USA) and quantified by Qubit (Invitrogen, Carlsbad, CA, USA). The amplicon pools were assessed for size and quantity using the Agilent 2100 Bioanalyzer (Agilent, Santa Clara, CA, USA) and the Illumina Library Quantification Kit (Kapa Biosciences, Woburn, MA, USA), respectively. The libraries were sequenced on the Illumina NovaSeq PE250 platform (LC-Bio) according to the manufacturer’s recommendations. Paired-end reads were assigned to samples by their unique barcodes and subsequently trimmed to remove the barcode and primer sequence. Paired-end reads were merged using Pear (v0.9.6) [49].

High-quality clean reads were generated by performing quality filtering on raw reads under specific conditions using fqtrim (v0.94). Chimeric sequences were subsequently filtered using Vsearch (v2.3.4) [50]. The denoising and length filtering were performed using the DADA2 algorithm in QIIME2 (v2019) [51]. This process generated Amplicon Sequence Variant (ASV) feature sequences and an ASV abundance table, with singleton ASVs (features appearing only once) removed.

Taxonomic annotation of ASV (feature) sequences was performed using the UNITE database (https://unite.ut.ee/). The abundance of each taxon in individual samples was quantified based on the ASV (feature) abundance table (Confidence threshold: 0.7).

### 2.4. Data Analyses

Diversity analysis of fungal communities was conducted using R software (v4.4.3). Specifically, the “vegan” package (v2.6.10) was employed for calculating the α-diversity indices, including Chao1, Shannon indices, and Simpson indices. Differences among groups were assessed using the Kruskal-Wallis test, followed by Dunn’s post hoc test. Venn diagrams were generated using the “venn” (v1.12) and “ggvenn” (v0.1.10) packages to visualize the shared and unique ASVs abundance among samples. Beta diversity was evaluated using non-metric multidimensional scaling (NMDS) based on Bray-Curtis and Jaccard distance matrices, implemented in R with the vegan package. Fungal co-occurrence networks at the phylum level were constructed based on Spearman correlations analysis (|R| > 0.7, *p* < 0.01) and visualized with Gephi software (v0.10.1). Fungal community functional prediction was performed using the “FUNGuildR package” (v0.3.0) in R (v4.4.3), based on the FUNGuild database (http://stbates.org/funguild_db.php, accessed on 28 November 2025). Predictions were filtered for reliability, retaining classifications with a confidence ranking of "Probable" or higher, and a clustered heatmap of the dominant functional guilds was generated using the “pheatmap” package (v1.0.13). Finally, redundancy analysis (RDA) was carried out using the vegan package in R (v4.4.3) to evaluate the correlations between fungal community structure and environmental factors.

## 3. Results

### 3.1. Distribution Characteristics of Environmental Factors in the Leizhou Peninsula Coastal Waters

**Figure 2:**
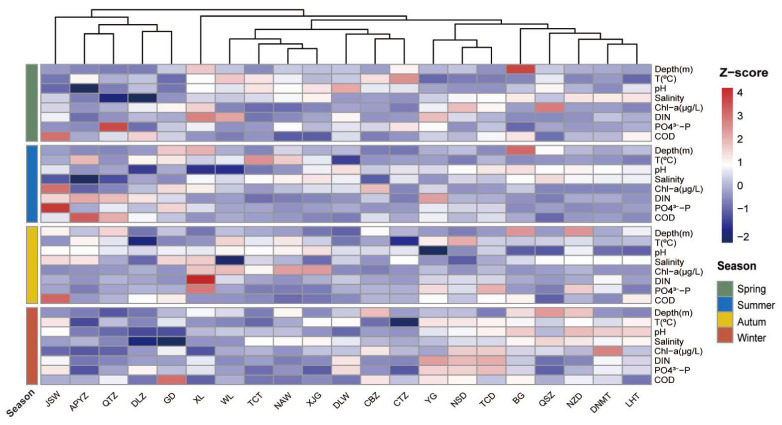
Heatmap of Z-score normalized physicochemical variables across 21 sampling stations in the coastal waters of the Leizhou Peninsula, China.

Physicochemical parameters from 21 sampling stations were normalized using Z-scores and visualized as a heatmap (Figure 2). The depth of sampling sites ranged from 2.3 m to 33.1 m across four seasons (Table S2). Water temperature varied significantly, with lower temperatures observed in spring and winter, and higher temperatures in summer and autumn (Table S2). The pH values were relatively stable, ranging from 7.31 to 8.90 (Table S2). Salinity ranged from 16.39 to 34.73, with a clear spatial gradient: salinity decreased from northern sites (e.g., QSZ, LHT, DNMT) towards the southern sites (e.g., CBZ, APYZ, QTZ), and also decreased from the bay entrance (e.g., QSZ) towards the inner bay (e.g., DLZ, QTZ) (Table S2). The concentration of DIN ranged from 0.02 to 1.43 mg·L^−1^ (Table S2). The concentration of SiO_3_^2−^-Si ranged from 0.04 to 3.90 mg·L^−1^, while PO_4_^3−^-P concentrations ranged from 0.01 to 0.29 mg·L^−1^ (Table S2). COD ranged from 0.32 to 4.00 mg·L^−1^ (Table S2). Spatially, concentrations of DIN, PO_4_^3−^-P, SiO_3_^2−^-Si, and COD generally increased from the southern peninsula to the northern sites (Table S2). Chlorophyll *a* (Chl *a*) concentration exhibited noticeable variation across the four seasons, with values at NAW ranging from 0.00 to 30.03 μg·L^−1^ (Table S2). Higher average Chl *a* concentrations were observed at southern bay-mouth stations (Table S2).

### 3.2. Composition of Fungi in the Leizhou Peninsula Coastal Waters

High-throughput sequencing of the fungal ITS region from the seawater samples collected along the Leizhou Peninsula, Zhanjiang, China, yielded a total of 7,002,633 raw reads, with an average of 83,365 ± 3502 reads per sample. After paired-end merging, quality filtering, and chimera removal, 6,060,479 high-quality sequences were retained (average 72,149 ± 5837 reads per sample), corresponding to a total of 1922.69 Mb of effective data. Denoising using the DADA2 pipeline generated 8777 ASVs across all samples, comprising 5,299,442 total reads.

**Figure 3:**
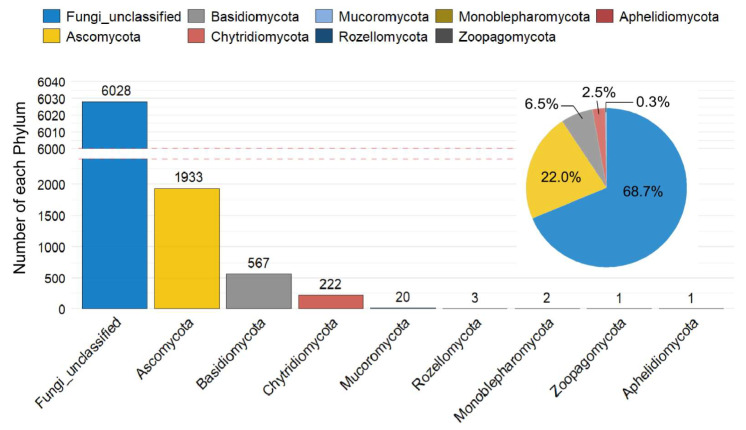
Fungal community composition at the phylum level based on ASV analysis in the Leizhou Peninsula, China.

**Figure 4:**
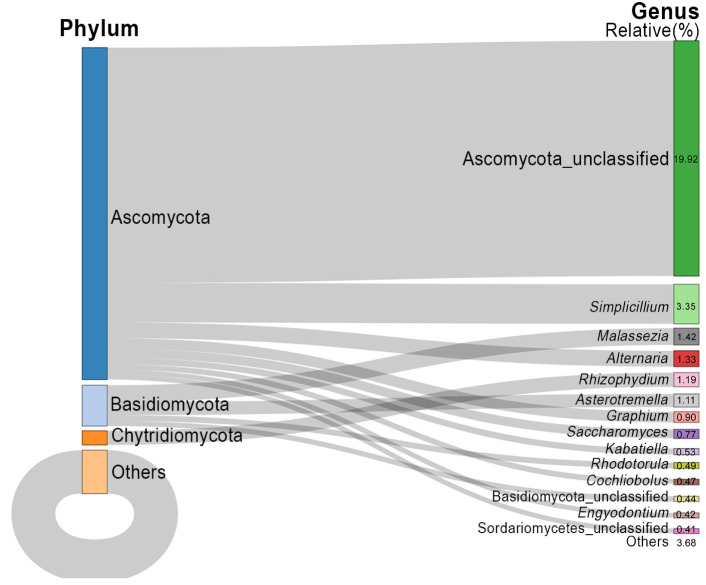
Relative abundance of the top 15 fungal genera in the Leizhou Peninsula, China.

In total, the recovered ASVs were taxonomically assigned to 10 phyla, 38 classes, 100 orders, 205 families, and 358 genera (Figure 3 and Figure 4). At the phylum level, a large proportion of sequences could not be confidently assigned to known fungal phyla (68.7%) (Figure 3). Among the classified taxa, Ascomycota was the dominant phylum, accounting for 22.0% of the total relative abundance, followed by Basidiomycota (6.5%) and Chytridiomycota (2.5%). The remaining phyla collectively contributed less than 0.3% (Figure 3).

After excluding taxa unclassified at the phylum level (Fungi unclassified, 68.7%) and those assigned to Ascomycota but unresolved at the class level (Ascomycota unclassified, 19.92%), the fungal communities were dominated by Sordariomycetes (6.50%) and Dothideomycetes (3.18%), both within the phylum Ascomycota. Species within Sordariomycetes were widely distributed and predominantly saprotrophic. Dothideomycetes represented the largest class within Ascomycota and included numerous plant pathogens.

At the genus level, the fifteen genera with the highest relative abundances were identified (Figure 4). After excluding taxa that could not be assigned to any phylum (68.7%), the top ten genera included Ascomycota unclassified (19.92%), *Simplicillium*, *Malassezia*, *Alternaria*, *Rhizophydium*, *Asterotremella*, *Graphium*, *Saccharomyces*, *Kabatiella*, and *Rhodotorula* (Figure 4). The most abundant genus, *Simplicillium*, belonged to the class Sordariomycetes and accounted for 3.35% of the total relative abundance. This was followed by *Malassezia* (1.42%), *Alternaria* (1.33%), and *Rhizophydium* (1.19%). Among the top ten genera, *Rhizophydium* was the only genus affiliated with Chytridiomycota, whereas the remaining genera belonged to Ascomycota and Basidiomycota. Species of *Rhizophydium* belonged to the class Chytridiomycetes and represented one of the earliest-diverging fungal lineages. They were predominantly aquatic and parasitized algae and other aquatic fungi.

### 3.3. Spatiotemporal Distribution of Fungi in the Leizhou Peninsula Coastal Waters

**Figure 5:**
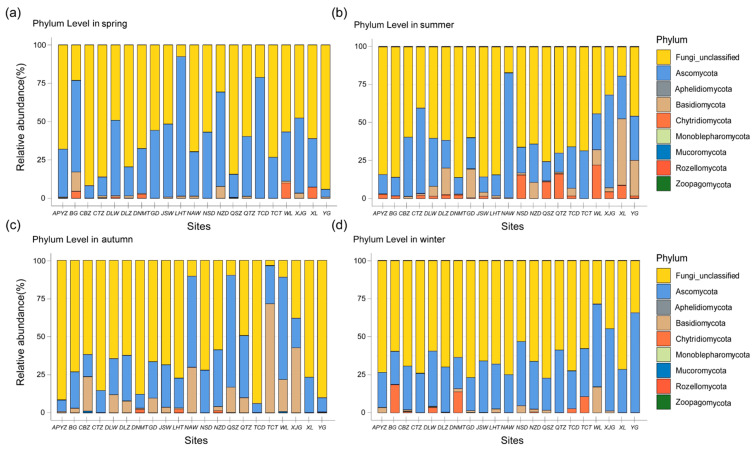
Species composition and relative abundance of fungal communities at the phylum level in coastal waters of the Leizhou Peninsula, China (**a**) spring; (**b**) summer; (**c**) autumn; (**d**) winter; The “unclassified” segment represents sequences that could not be assigned to a known phylum.

A percentage stacked bar chart was generated to illustrate the relative abundances of marine fungal communities at the phylum level in the coastal waters of the Leizhou Peninsula across four sampling seasons (Figure 5). Overall, unclassified fungal sequences (Fungi unclassified) dominated the communities at the phylum level and ranked first in relative abundance at most sampling stations across all four seasons. From summer onward, the proportion of unclassified fungi exhibited a gradual increasing trend, with a particularly pronounced increase observed in autumn. A notable exception was observed at station XJG. At this station, Basidiomycota dominated only in autumn, whereas Ascomycota exhibited the highest relative abundance during the other three seasons, contrasting with the general pattern observed at other stations. Across all sampled sites, Ascomycota, Basidiomycota, and Chytridiomycota consistently ranked as the three most abundant phyla. Chytridiomycota never exceeded a 5% relative abundance threshold in autumn and generally occurred at low proportions across all stations. Although Mucoromycota had a low average relative abundance (all <0.1%), it was detected in summer, autumn, and winter, whereas other rare phyla each accounted for less than 1% of the total community. Ascomycota exhibited the highest mean relative abundance in spring (Figure 5a), reaching up to 91% at station LHT, but displayed a comparatively lower abundance in summer (Figure 5b). Both Basidiomycota and Mucoromycota reached their highest average relative abundances in autumn, whereas Chytridiomycota was mainly observed in winter.

**Figure 6:**
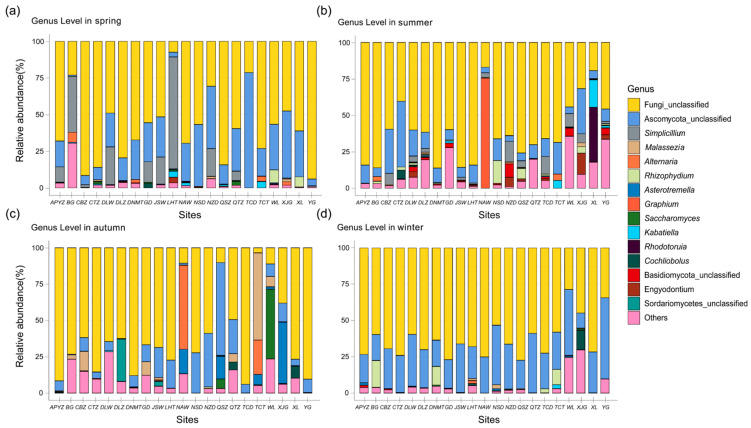
Genus-level composition and relative abundance of fungal communities in coastal waters of the Leizhou Peninsula, China. (**a**) spring; (**b**) summer; (**c**) autumn; (**d**) winter; (The “unclassified” segment represents sequences that could not be assigned to a known genus.).

The temporal compositional dynamics of marine fungal communities at the genus level across four seasons were shown in Figure 6. In spring, the fungal communities were dominated by Ascomycota unclassified and the genus *Simplicillium*, which predominated at most sampling stations. Specifically, station TCD exhibited the highest relative abundance of Ascomycota unclassified (78.66%), whereas station LHT was dominated by *Simplicillium*, accounting for 76.44% of the total community. Notably, the “Others” category accounted for the highest proportion at station BG (30.39%), followed by *Alternaria* (6.81%), a genus within Ascomycota. In addition, the fungal community at station BG displayed relatively high evenness, in contrast to stations such as LHT and TCD, which were strongly dominated by a single taxon. The Chytridiomycota genus *Rhizophydium* was most abundant at stations WL and XL, with relative abundances of 8.66% and 6.85%, respectively. The relative abundances of all remaining genera at each station were below 5%. In summer, fungal community richness increased compared with spring, with Ascomycota and the “Others” category dominating most sampling stations. Within Ascomycota, in addition to Ascomycota unclassified and *Simplicillium*, the relative abundances of *Graphium*, *Kabatiella*, and *Engyodontium* increased markedly. Notably, *Graphium* accounted for as much as 75.29% of the community at station NAW.

Compared with spring, the fungal community composition in summer was more diverse and was no longer dominated exclusively by Ascomycota. Taxa belonging to Basidiomycota and Chytridiomycota also exhibited increased prominence. For example, the basidiomycetous genus *Rhodotorula* reached a relative abundance of 37.58% at station XL, ranking as the third most dominant genus at that site, while Basidiomycota unclassified was detected at 15 sampling stations. The Chytridiomycota genus *Rhizophydium* accounted for 15.6% of the fungal community at station NSD, representing the most dominant classified genus at that site after unclassified taxa. In contrast, *Simplicillium*, which exhibited relatively high abundance in spring, showed a general decline in summer, accompanied by a pronounced shift in its spatial distribution pattern.

Autumn represented the peak period of fungal diversity, during which multiple genera belonging to both Ascomycota and Basidiomycota exhibited pronounced dominance across numerous sampling stations. The basidiomycetous genus *Asterotremella* was widely distributed, occurring at 16 stations including XJG (41.95%), NAW (16.90%), and QSZ (15.49%), and reached its highest relative abundances during this season. Within Ascomycota, *Malassezia* reached a relative abundance of 59.83% at station TCT, making it the most dominant ascomycetous genus in autumn. Although its relative abundance was low at some individual stations, *Malassezia* was detected at all stations except XJG and NSD, indicating a relatively broad distribution across the study area. In addition, several ascomycetous taxa dominated specific stations, including Ascomycota_unclassified at QSZ (64.08%), *Alternaria* at NAW (57.78%), *Saccharomyces* at WL (47.93%), and Sordariomycetes unclassified at DLZ (29.04%).

In winter, the overall richness of the fungal community declined markedly, with Ascomycota unclassified emerging as the dominant group, particularly at station YG, where it accounted for 55.87% of the total community. *Cochliobolus* reached a relative abundance of 13.44% at station XJG, representing a notable taxon at that site. The Chytridiomycota genus *Rhizophydium* exceeded 10% relative abundance at stations BG, DNMT, and TCT, indicating its continued presence under winter conditions. Overall, the fungal community structure in winter was structurally less complex, with dominance concentrated in fewer taxa.

### 3.4. Diversity of Fungal Communities in the Leizhou Peninsula Coastal Waters

#### 3.4.1. Seasonal Variation of Fungal Diversity

**Figure 7:**
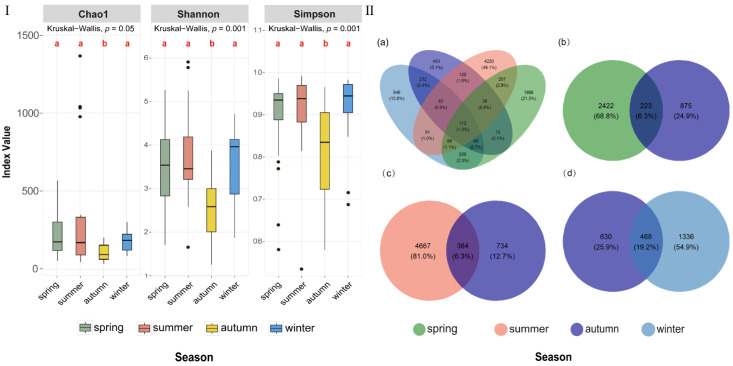
Alpha diversity and shared fungal ASVs across different sampling seasons in coastal waters of the Leizhou Peninsula, China. (**I**) Alpha diversity includes the Chao1 index, Shannon index, and Simpson index across four seasons. Different lowercase letters indicate significant difference (*p* = 0.001); black dots represent outliers. (**II**) Shared fungal ASVs across different sampling seasons: (**a**) four seasons; (**b**) spring VS. autumn; (**c**) summer VS. autumn; (**d**) winter VS. autumn.

α-diversity, reflecting species richness and evenness within an ecosystem, was evaluated using Chao1, Shannon, and Simpson indices. Significant differences among the four seasonal sample groups were assessed using the Kruskal-Wallis test followed by Dunn’s post hoc test (*p* = 0.001 or *p* = 0.05; Figure 7I).

The Chao1 index estimates the number of species in a community, including both observed and unobserved species. Species richness was highest in summer and lowest in autumn (*p* = 0.05), with spring and winter showing intermediate values.

The Shannon index, derived from information entropy, reflects both species richness and evenness, with higher values indicating greater diversity and more community structure. Shannon indices were significantly higher in spring, summer, and winter than in autumn (*p* = 0.001), indicating more even species distributions.

The 1-D Simpson index (Gini-Simpson) represents the probability that two randomly selected individuals belong to different species and is commonly used to assess community evenness and dominance by a few taxa. Kruskal-Wallis analysis revealed significant seasonal differences (*p* = 0.001), with Dunn’s post hoc comparisons showing significantly higher 1-D Simpson indices in spring, summer, and winter than in autumn. In autumn, 1-D Simpson indices ranged from 0.5798 to 0.9919, indicating considerable variation among sampling sites and that community structure at some sites was strongly dominated by a few taxa.

Overall, based on all three α-diversity indices, fungal diversity in autumn across the 21 stations was significantly lower than that in other seasons (*p* < 0.05), with no significant differences among spring, summer, and winter (*p* > 0.05). When combined with ASV-based Venn diagrams (Figure 7II), these indices further illustrated the similarities and differences among fungal communities. Figure 7II(a–c) showed that shared ASVs between autumn and spring, summer, and winter were 6.3%, 6.3%, and 19.2%, respectively, while unique ASVs in the other three seasons each exceeded 50%, confirming lower fungal diversity in autumn.

**Figure 8:**
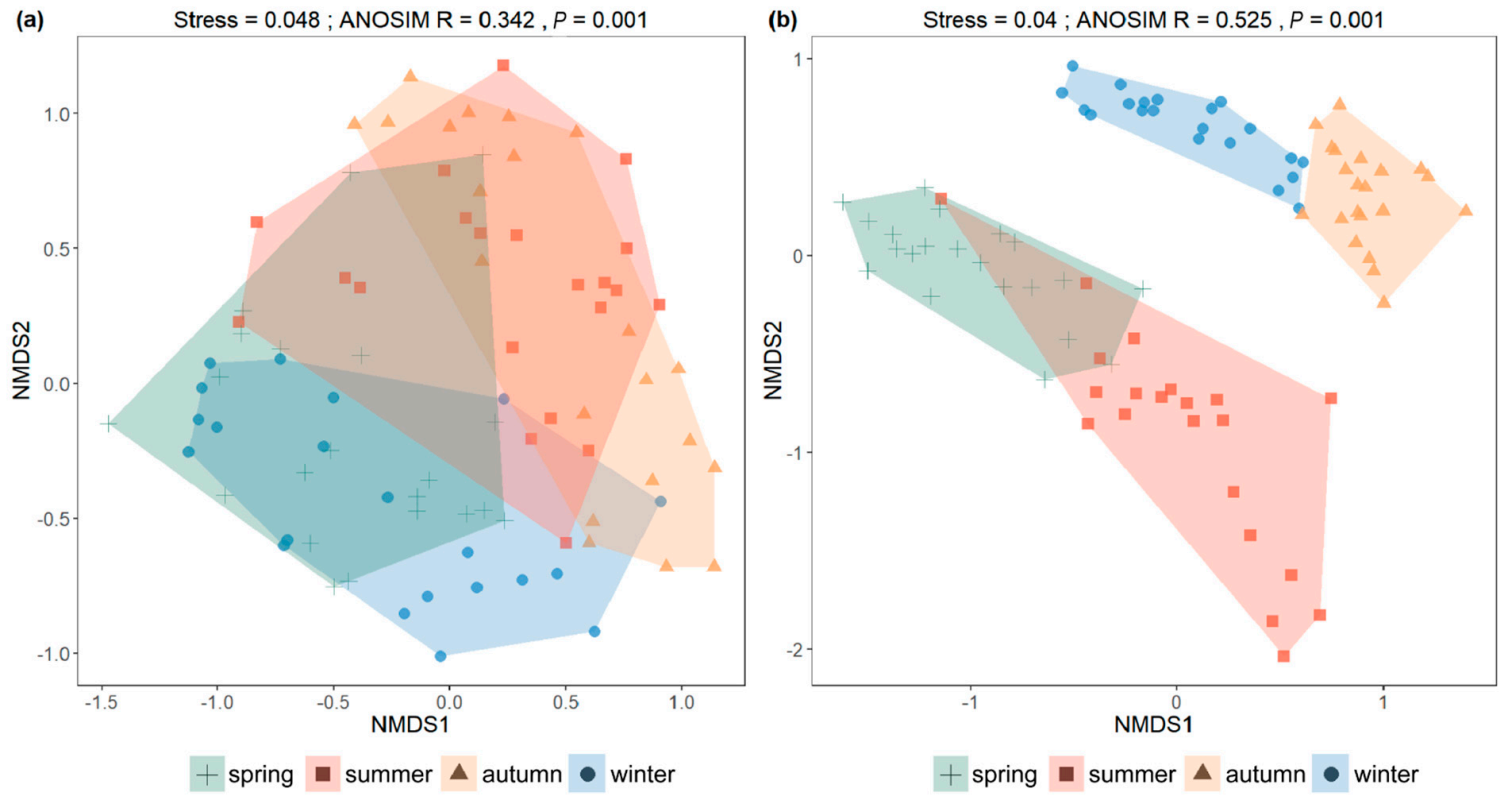
The β-diversity of fungal communities in coastal waters of the Leizhou Peninsula, China. NMDS analysis using (**a**) Bray-Curtis and (**b**) Jaccard distances.

Non-metric multidimensional scaling (NMDS) analysis based on Bray-Curtis and Jaccard distance matrices revealed significant seasonal differences in species composition and abundance (ANOSIM, *p* = 0.001; Figure 8). The two-dimensional stress values for both Figure 8a,b were below 0.05, indicating an excellent ordination fit and strong agreement with the original distance matrices. Figure 8a showed the NMDS ordination based on Bray-Curtis distances, incorporating both species abundance and presence/absence information. Although some overlap was observed, ANOSIM (*p* = 0.001) indicated significant inter-seasonal differences in species abundance. Figure 8b presented the NMDS ordination based on Jaccard distances, emphasizing species presence/absence. While slight overlap existed among seasons, the separation among seasonal groups was generally strong (0.75 > R > 0.5), indicating high seasonal species turnover.

Taken together, Bray-Curtis results indicated significant seasonal shifts in species abundance, despite partial overlap among samples. Overall, NMDS indicated that seasonal succession was a major driver of fungal community variation, reflecting both changes in species abundance and pronounced species turnover across seasons.

#### 3.4.2. Spatial Variation of Fungal Diversity Driven by DIN Gradients

**Figure 9:**
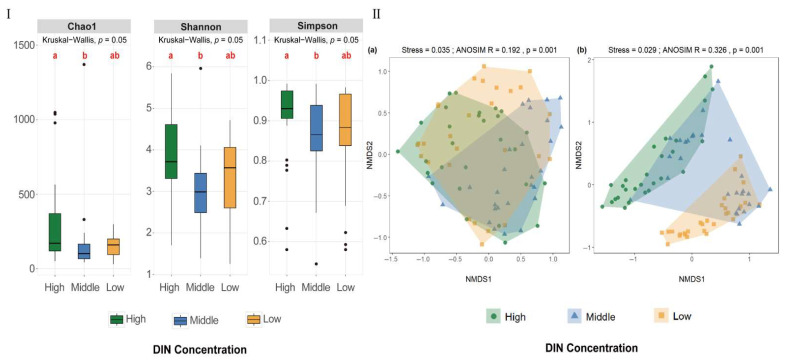
Alpha diversity and β-diversity of fungal communities across different DIN concentration groups in coastal waters of the Leizhou Peninsula, China. (**I**) Alpha diversity includes the Chao1 index, Shannon index, and Simpson index across different DIN concentration groups. Different lowercase letters indicate significant difference (*p* = 0.05); black dots represent outliers. (**II**) NMDS analysis using (**a**) Bray-Curtis and (**b**) Jaccard distances.

Based on the DIN gradient, the 21 sampling sites were categorized into low- (NAW, QSZ, TCT, CTZ, XJG, NZD, BG), medium- (GD, LHT, CBZ, APYZ, DLZ, QTZ, DLW), and high-DIN geographic regions (TCD, WL, DNMT, JSW, NSD, YG, XL), with seven sites included in each group. Figure 9I illustrated the α-diversity patterns of fungal communities across these DIN-defined regions, as assessed by the Chao1, Shannon, and Simpson indices.

All three α-diversity indices exhibited a consistent spatial trend along the DIN gradient. The high-DIN region showed the highest fungal diversity, whereas the medium-DIN region exhibited the lowest values, with significant differences observed between these two regions (*p* = 0.05). In contrast, no significant differences were detected between the low-DIN region and either the high- or medium-DIN regions. These results indicated that elevated DIN concentrations were associated with increased fungal diversity, reflected by higher species richness and evenness. The relatively low 1-D Simpson index observed in the medium-DIN region suggested a more uneven community structure, likely driven by the dominance of a limited number of taxa.

Furthermore, NMDS analysis based on Bray-Curtis and Jaccard distance matrices (Figure 9) showed that fungal communities differed significantly among high-, medium-, and low-DIN groups. The two-dimensional stress values were both below 0.05, indicating an excellent ordination quality and a high correspondence between the reduced-dimensional representation and the original dissimilarity structure (Figure 9II(a,b)). ANOSIM results for both distance matrices were highly significant (*p* = 0.001), suggesting statistically significant inter-group differences; however, the relatively small R values suggested limited separation among groups. Overall, the DIN gradient appeared to play an important role in structuring fungal communities.

### 3.5. Molecular Ecological Network Analysis of Fungal Communities

**Figure 10:**
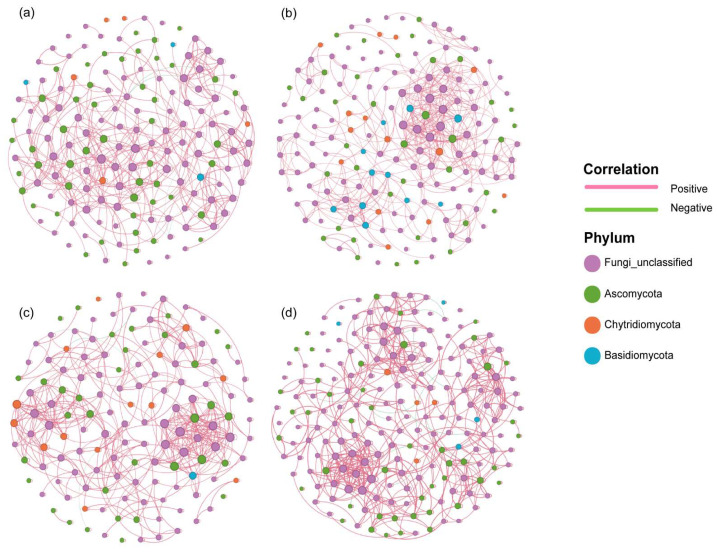
Molecular ecological network of fungal communities at the phylum level in the coastal waters of the Leizhou Peninsula, China. Each node represents a fungal phylum; size is proportional to its connectivity (degree) within the network. Connecting edges. (red for positive correlations, green for negative) represent strong and significant co-occurrence relationships. Panels (**a**–**d**) represent different sampling seasons. (**a**) spring, (**b**) summer, (**c**) autumn, (**d**) winter.

To explore the interactions among different fungal groups, co-occurrence networks were constructed for each sampling season: spring (Figure 10a), summer (Figure 10b), autumn (Figure 10c), and winter (Figure 10d) based on ASVs with a relative abundance threshold of more than 0.0175%. In these networks, nodes represented fungal phyla or unclassified ASVs detected in each season, and node size reflected their connectivity degree. Nodes were colored by phylum, with Ascomycota, Basidiomycota, and Chytridiomycota representing the dominant components. Edges represented statistically significant Spearman rank correlations between nodes, indicating potential co-occurrence or mutual exclusion relationships. Positive correlations (R > 0.6) were shown in red, whereas negative correlations (R < −0.6) were shown in green. Although many ASVs could not be classified at the phylum level, these nodes exhibited extensive connectivity and, together with classified taxa, formed a complex ecological interaction network. Therefore, unclassified nodes were retained to more comprehensively represent the overall fungal community structure.

Figure 10c depicts the co-occurrence network for autumn. This network comprised 141 nodes and 547 edges, with an average degree of 7.759. Although the network contained the fewest nodes and edges among the four seasons, it displayed the highest average degree, indicating relatively strong species interactions. The relatively short average path length (5.082) further indicated dense connectivity, while small modules (average clustering coefficient = 0.71, modularity = 0.737) suggested high local aggregation, pronounced modularity, and tightly connected subgroups. Positive correlations predominated the network, whereas negative correlations occurred mainly between Ascomycota and the other two phyla.

In spring, the network comprised 161 nodes and 546 edges, with an average degree of 6.783. The number of edges differed from autumn by only one, while autumn had 20 fewer nodes than spring. The higher average degree in autumn suggested more complex inter-node interactions. Additionally, network density and average clustering coefficient were higher in autumn than in other seasons, indicating that fungal interactions were most extensive and tightly connected in autumn.

In contrast, the winter network (Figure 10d) comprised the largest number of nodes and edges (203 nodes and 693 edges) but had a lower average degree (6.828), indicating that the increase in network size did not yield higher connectivity. Overall, the winter network was less dense; however, the modularity was high (0.775) and the average path length was the longest. Positive correlations predominated the network (98.27%), indicating multiple internally connected modules that were relatively distant and independent. Compared with autumn, the proportion of Basidiomycota nodes decreased, whereas Chytridiomycota nodes increased.

Under the same abundance threshold, the variations in node numbers across the four seasonal networks indicated that high-abundance and key functional taxa vary among seasons. Combined with the above results, this reflected seasonal dynamics and differentiation of fungal communities. Modularity in all four seasons’ networks was substantially greater than 0.3, indicating tightly interconnected communities, with nodes forming clustered “subgroup” topologies.

### 3.6. Functional and Trophic Characteristics of Fungi in Leizhou Peninsula Coastal Waters

**Figure 11:**
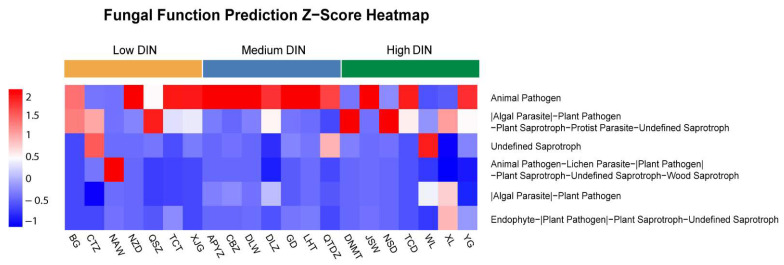
Z-score heatmap of the top 6 predicted fungal functions in coastal waters of the Leizhou Peninsula, China.

The FUNGuild database was employed to predict the potential functional profiles of fungal communities at 21 sampling sites across three DIN gradient regions. The abundance of each functional group at each site was normalized to a Z-score. The six most abundant functional groups were selected for visualization in a heatmap (Figure 11). Positive Z-scores indicated that a functional group’s abundance at a given site exceeded the mean of the top six groups, whereas negative Z-scores indicate below-average abundance.

The top six core functional groups were mainly dominated by pathogenic, parasitic, and saprotrophic strategies, with symbiotic strategies as a minor component. The pathogenic and parasitic categories included animal pathogens, algal parasites, plant pathogens, lichen parasites, and protist parasites. The saprotrophic category was primarily composed of plant saprotrophs, wood saprotrophs, and undefined saprotrophs. The symbiotic strategy was represented exclusively by endophytic fungi. Animal pathogens exhibited generally high abundance across all three DIN gradient regions and dominated all sites in the medium-DIN region, representing a functionally dominant group. Many functional groups displayed multifunctionality and relatively high Z-scores in both high- and low-DIN sites.

Overall, the composition of core functional groups across the seven medium-DIN sites was relatively uniform: animal pathogens were the dominant group, whereas other functional groups were below average, clearly differing from the high- and low-DIN regions.

### 3.7. Relationship Between Fungal Communities and Environmental Factors

**Figure 12:**
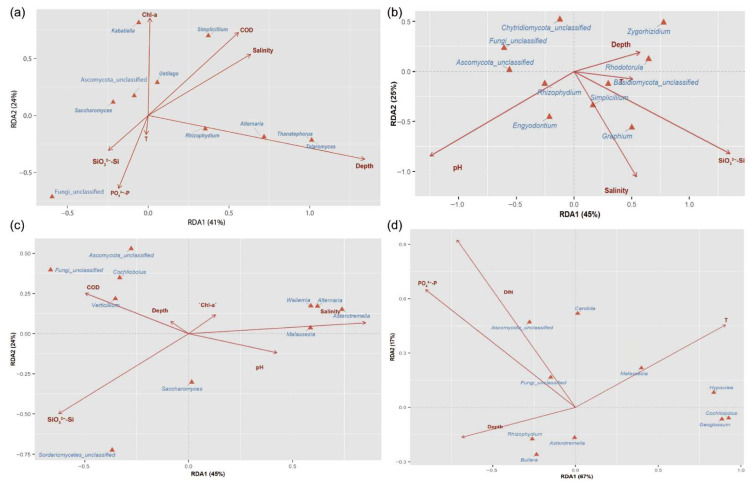
RDA of fungal community structure and environmental factors across the four sampling seasons in coastal waters of the Leizhou Peninsula, China (Redundancy analysis (RDA) biplot showing the relationship between the top 10 fungal genera, red triangle and blue labels: species; red vectors: environmental factors) (**a**) spring; (**b**) summer; (**c**) autumn; (**d**) winter.

Redundancy analysis (RDA) for spring (Figure 12a) revealed that seven environmental variables collectively explained 56.55% of the variation in fungal community structure (permutation test, F = 2.417, *p* = 0.0081), indicating that these factors were key drivers of community composition. Depth emerged as the most significant factor influencing community structure (permutation test, *p* = 0.005). RDA1 and RDA2 axes accounted for 41% and 24% of the total variance, jointly explaining 65%, indicating that the selected environmental variables captured most of the variation in fungal community composition. RDA1 represented the primary environmental gradient driving the fungal community structure in spring.

Along RDA1, vectors for Depth, COD, Chl *a*, and salinity pointed in the positive direction, indicating consistent spatial variation. This represented an environmental gradient from low-salinity, shallow waters with low chlorophyll to high-salinity, deeper waters with high chlorophyll along RDA1. SiO_3_^2−^-Si, PO_4_^3−^-P, and T pointed in the negative direction along both axes, indicating inverse correlations with other environmental factors. The small angle between SiO_3_^2−^-Si and PO_4_^3−^-P vectors indicated a close relationship, potentially reflecting a shared nutrient source at the sampling sites. The short vector for T suggested a minimal influence on fungal community structure.

Integration of RDA and Spearman correlation analyses identified relationships between fungal genera and environmental variables. Genera including *Thanatephorus*, *Talaromyces*, *Alternaria*, and *Rhizophydium* showed positive correlations with Depth. *Simplicillium* and *Kabatiella* were strongly associated with Chl *a*, COD, and salinity. *Kabatiella* exhibited a significant positive correlation with Chl *a* (r = 0.75, *p* = 7.1 × 10^−5^), and *Simplicillium* positively correlated with COD. *Ustilago* was positively correlated with salinity and COD but negatively correlated with SiO_3_^2−^-Si and PO_4_^3−^-P; the significant negative correlation with PO_4_^3−^-P (r = −0.60, *p* = 0.0041) suggested that *Ustilago* might preferentially inhabit lower-nutrient areas.

Redundancy analysis (RDA) of the top ten fungal genera in summer (Figure 12b) revealed that five environmental variables collectively explained 34.73% of the variation in community structure (permutation test, F = 1.5966, *p* = 0.0257). SiO_3_^2−^-Si and salinity emerged as the key drivers of community composition, showing highly significant effects (permutation test, *p* < 0.01). The RDA depicted relationships between environmental variables and fungal genera. The first two axes (RDA1 = 45%, RDA2 = 25%) jointly explained 70% of the variation, consistent with that in spring, with RDA1 remaining the primary driver of structural differences.

In the RDA biplot, pH exhibited the longest vector, pointing in the negative direction of RDA1, followed by two strongly collinear variables, SiO_3_^2−^-Si and salinity, both oriented along the positive direction of RDA1. Spearman correlation analysis revealed that *Engyodontium* and *Rhizophydium* showed significant positive correlations with pH (r = 0.49, *p* = 0.025; r = 0.63, *p* = 0.002), whereas *Zygorhizidium* demonstrated a significant negative correlation with pH (r = −0.67, *p* = 8.5 × 10^−4^). These results suggested that *Engyodontium* and *Rhizophydium* might be associated with more acidic environments, potentially reflecting acid tolerance or involvement in the degradation of acidic humic substances. *Simplicillium* and *Graphium*, both belonging to Ascomycota, exhibited notable adaptability to salinity. Their association with high-salinity and high-silicate environments suggested potential symbiotic or parasitic interactions with plants, particularly diatoms, under these conditions.

For autumn, the RDA biplot (Figure 12c) showed that six environmental variables together explained 38.98% of the total variation in fungal community composition (permutation test, F = 1.4906, *p* = 0.084). Salinity and SiO_3_^2−^-Si were identified as the most influential factors shaping community structure (permutation test, *p* < 0.01). RDA1 accounted for 45% of the explained variation, with salinity, pH, and COD strongly associated with this axis, among which only salinity showed a positive correlation. RDA2 explained 24% of the variation, and SiO_3_^2−^-Si, Depth, and Chl *a* were strongly associated with this axis, with SiO_3_^2−^-Si exhibiting a negative correlation. The biplot demonstrated clear separation of fungal genera along environmental gradients, with genera associated with similar environmental conditions clustering together. This pattern indicated niche differentiation driven by environmental heterogeneity, whereby taxa with similar ecological requirements or shared resources tended to aggregate, whereas others were spatially separated.

Some genera, including *Cochliobolus* and *Verticillium*, were positively correlated with COD and clustered in the upper-left portion of the biplot. *Asterotremella*, *Wallemia*, and *Alternaria* also showed positive correlations with COD. *Asterotremella* is a halotolerant, marine saprotroph that is associated with macroalgae, and its distribution varied along salinity gradient. *Saccharomyces* exhibited a positive correlation with SiO_3_^2−^-Si, suggesting a potential association with diatom-dominated conditions. *Cochliobolus* and *Verticillium* are typical saprotrophic decomposers capable of degrading organic detritus, which was consistent with their aggregation in areas with elevated COD.

The RDA biplot for winter (Figure 12d) showed that four environmental variables together explained 28.77% of the total variation in fungal community composition (permutation test, F = 1.6153, *p* = 0.045). RDA1 accounted for 67% of the variance, while RDA2 explained 17%, with a combined explanation of 84%, indicating that environmental factors exerted a stronger influence on fungal community structure in winter than those in other seasons. Temperature, DIN, phosphate, and depth were identified as the key drivers of community variation. Notably, the core environmental factors differed from those in the previous seasons, with DIN emerging as the most significant factor affecting community structure (permutation test, *p* < 0.01). High-nutrient areas characterized by elevated phosphate and DIN concentrations were associated with shallower depths, indicating that nearshore waters tended to contain higher nutrient levels. This pattern may reflect anthropogenic influences, as terrestrial inputs can increase nutrient concentrations in coastal waters.

## 4. Discussion

### 4.1. Community Composition and Taxonomic Patterns of Planktonic Marine Fungi in the Leizhou Peninsula Coastal Waters

Owing to the combined influences of terrestrial input, hydrodynamic forcing, and water mass mixing, the waters of the Leizhou Peninsula display marked spatiotemporal heterogeneity in their spectral characteristics. Such complexity underscores the highly dynamic physicochemical status of this coastal ecosystem [52]. The Leizhou Peninsula encompasses diverse marine habitats, including shallow seas, deep-water regions, coral reefs, and seagrass beds. However, research on fungal diversity within the Zhanjiang area has predominantly focused on mangroves, intertidal zones, and marine sediments, leaving the fungal communities of coastal waters relatively under-explored.

In this study, ITS2 high-throughput sequencing was employed to characterize fungal communities in the coastal waters of the Leizhou Peninsula, Zhanjiang, China. From 84 samples, we identified a total of 8777 ASVs, spanning 10 phyla and 358 genera. Ascomycota exhibited overwhelming dominance, followed by Basidiomycota and Chytridiomycota (excluding the unclassified taxa). These results align with previous findings regarding planktonic fungal compositions in coastal waters.

Sen et al. [18] demonstrated that Ascomycota and Basidiomycota consistently dominate fungal communities across various oceanic regions. This pattern holds true irrespective of the methodology employed, whether through fungal isolation from deep-sea and subsurface sediments or via ITS-based sequencing of water and sediment samples [18]. Furthermore, a global meta-analysis of coastal water fungal 18S rRNA gene (V4 region) amplicon data, sourced from the Meta PR2 database [1], revealed a pronounced geographic bias in sampling efforts. Specifically, coastal habitats in the Southern Hemisphere remain significantly underrepresented in current mycological research. In the Northern Hemisphere, coastal fungal communities are primarily dominated by Ascomycota (36.6%), Chytridiomycota (33.4%), and Basidiomycota (19.3%). The taxonomic composition and relative abundance of these major groups are highly consistent with the observations in our current study. On a global scale, while a wide array of fungal phyla are represented within planktonic marine communities, Ascomycota, Basidiomycota, and Chytridiomycota emerge as the predominant groups [53]. Their taxonomic richness is likely a reflection of their deep evolutionary histories and efficient dispersal mechanisms across oceanic environments.

This community composition pattern is ubiquitous across global coastal ecosystems and aligns with previous observations from the Leizhou Peninsula and its adjacent waters. Regardless of the methodology—whether utilizing culture-dependent isolation or high-throughput sequencing—Ascomycota and Basidiomycota are invariably identified as the predominant phyla. An investigation into the enzymatic potential of intertidal fungi revealed that strains isolated from the Xuwen Coral Reef Nature Reserve (Zhanjiang) exhibited distinct taxonomic distributions based on their bioactivity. Strains capable of producing extracellular plasminogen activators were restricted exclusively to Ascomycota. In contrast, those secreting fibrinolytic-like enzymes were represented by both Ascomycota and Basidiomycota [36]. Likewise, Gao et al. [40] observed that in Zhanjiang Bay sediments across salinity gradients, Ascomycota and Basidiomycota jointly accounted for over 75% of the fungal community. Notably, 23.01% of the sequences remained taxonomically unassigned at the phylum level, reflecting the limitations of current reference databases in capturing marine fungal diversity. At a broader regional scale, fungal communities in areas including the Maowei Sea in the Beibu Gulf, Gouqi Island in Zhoushan Archipelago, and the Yangtze River Estuary are consistently dominated by Ascomycota and Basidiomycota [54,55,56]. However, a substantial portion of the fungal diversity remains taxonomically unresolved. For instance, approximately 31.4% of the sequences from the surface waters of Gouqi Island were unclassified at the phylum level. Notably, the proportion of unclassified fungi observed in the Yangtze River Estuary was lower than the 68.7% detected in our current study [55,56].

The substantial proportion of taxonomically unassigned sequences observed in this study is a phenomenon frequently documented in similar marine environments. This finding underscores the existence of a vast, yet-to-be-characterized ‘fungal dark matter’ within the coastal waters of the Leizhou Peninsula, suggesting a reservoir of novel phylogenetic lineages [19,57]. Notably, a stringent sequence quality control and taxonomic annotation strategy were implemented. By referencing the UNITE database and applying a high confidence threshold, we prioritized data reliability and minimized potential technical noise or spurious assignments that could otherwise bias the ecological interpretations. These results likely underscore the inherent limitations of current public reference databases for marine fungal ITS sequences. Previous studies have highlighted that the UNITE database exhibits significant geographic and habitat-specific biases, as its records are primarily derived from terrestrial ecosystems with sparse coverage of marine environments [58,59]. Consequently, the high prevalence of unclassified sequences suggests a substantial reservoir of unknown fungal taxa in the coastal waters of the Leizhou Peninsula. This representational gap reinforces the widely accepted consensus that “the number of described fungal species remains far below the actual diversity” [32,33]. Within this framework, these unassigned taxa may represent ecologically active but poorly characterized lineages, whose dynamics likely underpin the observed temporal restructuring of the community.

### 4.2. Seasonal Timing of Species Distribution in the Leizhou Peninsula, Zhanjiang, China

The unique physiological and biochemical traits of fungi enable them to adapt to significant seasonal environmental shifts, which constitute a key ability for occupying ecological niches in aquatic ecosystems [60,61]. Specifically, seasonal fluctuations in water temperature, pH, and Chl *a* concentrations serve as primary determinants of fungal community structure, driving the pronounced spatiotemporal heterogeneity observed across coastal waters [62,63].

The abundance and diversity of coastal fungal communities undergo distinct seasonal transitions in response to fluctuating environmental variables [63,64,65]. Our results demonstrated a clear seasonal succession in the Leizhou Peninsula’s coastal waters, with dominant phyla exhibiting specific temporal preferences. One-way ANOVA revealed that seasonality significantly influenced the abundance of Basidiomycota and Chytridiomycota (*p* < 0.001), while Ascomycota maintained a stable and overwhelming dominance (27.47–38.22% of the classified sequences) across all seasons. Post-hoc SNK tests further specified that Basidiomycota reached peak abundance in autumn, significantly exceeding levels in winter and spring (*p* < 0.05). Conversely, Chytridiomycota abundance peaked in summer, significantly surpassing that in autumn (*p* < 0.05). These findings reflected a dynamic temporal turnover: while Ascomycota remained a year-round core constituent, phyla such as Basidiomycota, Mucoromycota, and Chytridiomycota exhibited pronounced seasonal pulses. This pattern aligns with observations from the Piver’s Island Coastal Observatory (PICO), where Ascomycota dominance persists year-round alongside the seasonal fluctuations of secondary fungal lineages [64].

In addition to the phylum-level seasonal distribution, the α- and β-diversity analyses, as well as the phylum-level co-occurrence network, further demonstrated a clear seasonal succession of fungal communities in the coastal waters of the Leizhou Peninsula. Analyses of the Shannon, Simpson, and Chao1 indices showed that the diversity of fungal communities in autumn was significantly lower than that in the other three seasons. Although no significant differences were observed among spring, summer, and winter, seasonal fluctuations in community structure were evident. Additionally, NMDS analyses based on Bray-Curtis and Jaccard distance matrices showed clear clustering of community samples across different seasons, indicating that seasonality was the primary driver of community composition changes. ANOSIM analysis further indicated that species turnover was the main mechanism responsible for seasonal differences, while changes in species abundance served as secondary drivers [66].

The α-diversity analysis revealed seasonal succession in fungal community structure, while β-diversity analysis further confirmed that seasonal changes were the key force shaping the community structure. These statistical results not only confirmed the existence of seasonal succession but also provided evidence for exploring the roles of environmental and biotic factors in driving community structure.

Seasonal changes can be divided into environmental and non-environmental variations. Seasonal environmental changes serve as an important natural selective pressure that likely drives local adaptation of fungal communities, leading to species turnover and abundance changes [67]. In coastal waters, seasonal environmental changes are primarily driven by climate, hydrology, and geography, which include periodic water mass transport, riverine input, tidal mixing, and wind forcing [68]. Specific environmental factors such as nutrients, temperature, pH, salinity, and chlorophyll exhibit seasonal variations that significantly affect fungal community composition [63,64,69]. Mechanistically, environmental factors such as temperature influence fungal growth, survival, reproduction, and dispersal [70], and fungal adaptation to these factors also affects intraspecific variation and species distribution [71].

Additionally, local adaptation can be shaped by both biotic and abiotic factors [72,73], and such adaptation is likely to alter the interactions between fungi and other organisms, thus influencing their ecological niche, adaptability, and community distribution. Phylum-level co-occurrence network analysis further supports this conclusion: network diagrams for the four seasons showed differences in node connectivity, network density, and the centrality of key phyla. For example, Ascomycota occupied a central hub in the network during spring, autumn, and winter, while Basidiomycota and Chytridiomycota were more active in summer and autumn, which indicates that different fungal phyla exhibit distinct ecological roles and interaction patterns across seasons [74].

In autumn, the co-occurrence network exhibited high modularity, high clustering, and low overall connectivity, with high local connectivity. However, its diversity and community evenness were the lowest among the four seasons. Combined with the RDA for autumn, the results showed that the measured environmental variables explained a minor proportion of the community structure (permutation test, *p* = 0.084), suggesting that other factors may ominate community distribution [75]. The high modularity, high clustering, and high complexity of the phylum-level network indicated a high degree of organization among species within the community. This supports the hypothesis that biotic interactions drive the heterogeneity in community distribution and emphasizes the importance of considering biotic factors in fungal community assembly research, while also reminding researchers not to overlook the impact of biotic factors on community construction. In the co-occurrence network, nodes representing “Unclassified Fungi” were relatively abundant, with some nodes exhibiting high centrality, suggesting that these “fungal dark matter” taxa may be activated in specific seasons [19].

### 4.3. Mechanistic Insights into Nutrient-Driven Fungal Community Assembly

These findings highlight seasonal fluctuations as a fundamental environmental filtering mechanism. By exerting periodic selective pressure at a macro-ecological scale, these temporal shifts dictate the assembly of fungal groups, ultimately driving a robust and predictable seasonal succession in community architecture on an interannual basis [76]. The spatial partitioning of the study area based on DIN concentrations is strongly corroborated by established scientific evidence, reflecting the critical role of N availability in shaping coastal ecological zones. A long-term observational and statistical analysis of Zhanjiang Bay from 2017 to 2021 demonstrated a highly significant positive correlation between the Eutrophication Index (EI) and DIN (r = 0.794, *p* < 0.01) [77]. Furthermore, DIN serves as an integral component of the internationally recognized EI framework and is widely utilized to quantify nutrient loading and assess the trophic status of coastal waters. Building upon this rationale, the 21 sampling sites across the Leizhou Peninsula were categorized into three distinct regions along a nutrient gradient, primarily defined by their respective DIN concentrations. Various statistical analyses revealed that the geographic spatial differentiation based on DIN gradients is independent of seasonal succession, acting as a relatively independent local driver, leading to spatial heterogeneity in fungal community structure across the sampling regions of the Leizhou Peninsula [78].

The NMDS analysis demonstrated pronounced spatial variation in the β-diversity of fungal communities across different DIN gradients. Specifically, fungal community structures in areas with varying DIN levels were distinctly separated in the ordination space, indicating notable differences in community composition among waters with different DIN concentrations. These findings imply that DIN is a primary factor in shaping the observed spatial heterogeneity of fungal communities in the coastal waters of the Leizhou Peninsula. Subsequently, the α-diversity analysis further indicates that the species abundance of fungal communities is closely related to the DIN gradient. The three α-diversity indices (Chao1, Simpson, and Shannon) revealed a consistent pattern: the Chao1 index indicated that species abundance was highest in high-DIN waters, followed by low-DIN and then medium-DIN regions, with significant differences observed specifically between high- and medium-DIN sites. To further investigate this trend, we performed Spearman correlation analysis between DIN concentration and the α-diversity indices (Figure S1). The analysis revealed a highly significant positive correlation with the Chao1 index (R = 0.7, *p* = 0.00059), suggesting that fungal species richness tends to increase with rising DIN levels across the studied waters. This finding aligns with previous research in Zhanjiang Bay, Guangdong, which reported a positive correlation between fungal richness in surface seawater and inorganic nitrogen concentrations based on linear regression analysis [79]. The positive correlation observed is broadly consistent with the historical trend reported in Zhanjiang Bay [73]. However, our boxplot analysis revealed a non-linear response in the Chao1 index to the DIN gradient, with the lowest species richness occurring at medium, rather than low, DIN concentrations. This non-linear pattern suggests the activation of distinct community assembly mechanisms in regions with medium DIN concentrations. According to the classical competitive exclusion principle, species with similar ecological niches cannot coexist stably under resource-limited conditions [80]. Specifically, in contrast to the oligotrophic conditions of low-DIN waters, intermediate nutrient enrichment may diminish the competitive edge of specialized K-strategists adapted to nutrient scarcity. This shift allows opportunistic species to proliferate in response to the increased resource availability. By rapidly exploiting these resources and occupying available niches, they can outcompete other functional groups and specialized taxa, ultimately leading to a net decrease in overall species richness. This pattern was corroborated by the Shannon and Simpson indices, which revealed a synchronous decline in community evenness within medium-DIN regions. These indices confirmed the lowest diversity levels at intermediate concentrations, where community structure became dominated by a few taxa [19,81,82].

Based on the predefined DIN gradient (high, medium, low), we predicted the core functional guild composition of the fungal communities. This analysis revealed significant differences in functional structure across the three nutrient regimes. In medium-DIN regions, the functional structure exhibited a highly simplified pattern, characterized by the dominance of “animal pathogens” as the core functional guild. Even when examining the top six functional groups, the relative abundance of non-pathogenic guilds remained consistently low, a pattern that was remarkably uniform across all seven sampling sites. This finding corroborates our earlier hypothesis: in medium-DIN regions, nutrient input may drive the rapid proliferation of animal pathogens by increasing host abundance and elevating infection risk. The dominance of pathogenic groups likely creates competitive pressure that inhibits the coexistence of other fungal functional guilds, ultimately driving the observed decline in both species richness and functional diversity [19]. When a single functional group achieves dominance, the rapid proliferation of opportunistic species and their preemption of available resources can compress the ecological niches of other functional groups, ultimately driving community diversity to its lowest level. This distribution pattern is likely shaped by local geographical selection and diffusion limitations. First, spatial heterogeneity in key environmental factors—particularly nutrient levels—across the Leizhou Peninsula imposes differential selective pressures on fungal communities. Continuous environmental selective pressure drives the coevolution of fungal infectivity and host defense, forming specialized interaction networks. In contrast, regions lacking strong biotic interaction pressure may exhibit different interaction patterns between fungi and hosts, a phenomenon consistent with the “geographic mosaic coevolution theory” [19,83]. Furthermore, observed spatial differences may be partially attributed to diffusion limitations, with waters possessing similar hydrological characteristics possibly acting as hydrological barriers, further reducing the likelihood of species exchange due to the inherent diffusion limitations of fungi [84]. PERMANOVA analysis further confirmed significant differences in functional structures across regions with different DIN gradients (R^2^ = 0.8884, *p* < 0.001), indicating that DIN gradients explain 88.84% of fungal functional variation. Pairwise comparisons (FDR-corrected) revealed highly significant differences between all DIN gradient levels: High vs. Low (R^2^ = 0.749, *p*. adj < 0.001), Low vs. Medium (R^2^ = 0.923, *p*. adj < 0.001), and High vs. Medium (R^2^ = 0.749, *p*. adj < 0.001). Based on the above results, it can be concluded that DIN levels are the primary environmental factor driving the functional structure differentiation of fungal communities in the coastal waters of the Leizhou Peninsula.

The coastal zone is situated in the land–sea transition zone, representing a typical estuarine ecosystem. Due to the varying types and distributions of human activities, along with the uneven spatial distribution of industrial and agricultural development, the coastal zone exhibits spatial heterogeneity in development, nutrient input, and disturbance [85,86]. Analyzing the background of human activity in Leizhou Peninsula sampling sites, it is evident that aquaculture areas dominate across different DIN gradients, though the water environmental characteristics differ significantly. Specifically, the low-DIN regions included five aquaculture sites, one industrial site, and one coastal tourism site, most of which are located in water bodies directly exposed to the open sea, with frequent water exchange that prevents nutrient accumulation over time [87]. The high-DIN areas comprised four aquaculture sites and three tourism sites, all located in bays or semi-enclosed waters, with no industrial sites included. Although terrestrial inputs in these areas may not be particularly intense, the limited water exchange and prolonged retention times typical of such enclosed environments likely facilitate nutrient accumulation, accounting for the elevated DIN levels. In contrast to both high- and low-DIN sites, the medium-DIN sites were primarily located in transitional areas characterized by more complex hydrodynamic conditions. These sites, predominantly in semi-enclosed water bodies with limited direct exposure to the open sea, receive terrestrial nutrient inputs while maintaining partial water exchange capacity. This creates a continuously fluctuating, non-accumulative nutrient regimes—an inherently unstable environment. Building on the preceding discussion, we hypothesize that such nutrient instability intensifies intra-community competition, favoring opportunistic species and ultimately leading to a more homogeneous community structure with reduced species richness.

### 4.4. Hypothesis of Dual-Driving Mechanisms

Community assembly is shaped by multiple ecological processes, and the underlying mechanisms are often interpreted through two complementary theoretical frameworks: niche-based processes and neutral processes [88,89]. Integrating temporal (seasonal) and spatial (DIN gradient) analyses, this study proposes a “dual-driving mechanism” for fungal community assembly along the Leizhou Peninsula. This hypothesis posits that community structure is shaped by the interplay between seasonal succession and local nutrient-driven effects, rather than by these factors operating independently [90]. Classical ecological theory emphasizes priority effects, competition-colonization trade-offs, and dispersal limitation as key determinants of community structure [91,92].

This study further revealed that the dominant role of DIN exhibits pronounced seasonal characteristics, most evident in the winter RDA. During this season, DIN emerged as the most significant environmental factor structuring community composition (permutation test, *p* < 0.01), with its vector showing a nearly orthogonal orientation to that of seasonal factors such as water depth and temperature. This suggests that during winter, the relative influence of typical seasonal factors—such as water temperature and depth—diminishes, allowing DIN to emerge as the primary selective pressure that filters fungal taxa [93,94]. Moreover, under winter conditions, taxa possessing functional traits adapted to local nutrient regimes (DIN) are more likely to be preferentially selected and establish dominance [95]. These early colonizers establish themselves through competitive exclusion, niche occupation, and modification of biotic interactions. In doing so, they shape specific microenvironments that lay the foundation for the core functional groups persisting throughout the year [96]. For example, in this study, low-DIN waters were characterized by functional groups such as animal pathogens and parasitic fungi; medium-DIN regions were overwhelmingly dominated by a single guild—animal pathogens; while high-DIN regions supported a more diverse functional assemblage, including animal pathogens, algal parasites, and saprotrophs. These early colonizers establish priority effects by occupying key ecological niches. Even as seasonal environmental conditions shift, the community maintains similar core functions due to the combined influence of priority effects and functional conservatism, resulting in the relative stability of DIN gradient-associated functional structures [91]. Additionally, statistical analysis revealed significant differences in fungal community structure across DIN levels, while within-group dispersion remained remarkably consistent (average within-group distances: high-DIN = 10.56, medium-DIN = 10.57, low-DIN = 10.56). This result indicates that communities within each DIN level maintained relatively stable and distinct configurations, further validating that the observed differences reflect genuine shifts in community composition rather than heterogeneous within-group variability.

### 4.5. Future Perspectives

Synthesizing our findings, this study demonstrates that fungal communities in the coastal waters of the Leizhou Peninsula are shaped by clear drivers operating at both temporal and spatial scales. Time functions as a key external environmental factor structuring these communities, while DIN exerts significant influence across spatial gradients. Collectively, these results highlight the sensitivity of coastal marine fungi to anthropogenic environmental changes, while also revealing their capacity to maintain relatively stable community configurations under specific nutrient regimes—underscoring their vital ecological role in complex coastal ecosystems.

As mentioned above, this study employed ITS2 amplicon sequencing to analyze coastal samples from the Leizhou Peninsula, and the results showed that 68.7% of the recovered ASVs could not be classified at the phylum level. This outcome is primarily attributed to the limitations inherent in amplicon-based techniques or the presence of potential novel species within the samples that have not yet been fully identified or described, thus preventing classification into any known phyla in the reference databases. To address this methodological limitation, future diversity studies should prioritize the use of metagenomic sequencing. Compared to amplicon sequencing, metagenomics offers a higher resolution, overcoming biases such as primer mismatches and primer preferences, thus preventing the underrepresentation of important taxa [97,98]. Additionally, metagenomics allows for the elucidation of key metabolic pathways involved in the degradation of marine-derived biopolymers (e.g., chitin, alginate, cellulose) and N source assimilation, further emphasizing the ecological importance of fungi in material cycling and biogeochemical processes [99,100,101]. The study of fungal diversity in coastal waters offers valuable insights into the impacts of human activities on microbial community dynamics, thereby providing a scientific basis for environmental management strategies. Marine fungi, though ecologically important, have long been a neglected component of coastal ecosystems. They now face mounting threats, including habitat alteration and degradation, biological invasions, climate change, species extinction, population decline, and the erosion of critical ecosystem functions [19,102,103]. Existing research suggests that mitigating these impacts requires targeted measures such as regulating nutrient and pollutant inputs, controlling the introduction of invasive species, and protecting key habitat structures—approaches that have proven effective in safeguarding aquatic microbial communities [102].

Therefore, future research on coastal aquatic fungal diversity and community structure should be integrated with regional environmental characteristics and local human activity contexts. Such context-specific investigations are essential for developing robust assessment methods and adaptive management strategies. This research direction will not only help fill critical knowledge gaps in marine fungal ecology but also provide the scientific foundation for more effective, targeted conservation measures in coastal ecosystems.

## 5. Conclusions

This study represents the first systematic investigation of fungal communities in the coastal waters of the Leizhou Peninsula, China, using ITS2 sequencing. The fungal community was predominantly composed of Ascomycota, Basidiomycota, and Chytridiomycota, with Ascomycota exhibiting absolute dominance across all seasons and sampling sites. Clear seasonal dynamics were observed: species richness peaked in autumn (August), while evenness reached its minimum in autumn, when communities became dominated by a few abundant taxa. Co-occurrence network analysis further revealed tightly connected nodes and highly modular structures in autumn assemblages.

Superimposed on these temporal patterns were pronounced spatial variations driven by dissolved inorganic nitrogen (DIN) gradients. High-DIN regions exhibited the highest species richness, while low-DIN areas showed the lowest evenness. Both NMDS and ANOSIM analyses confirmed DIN as a key factor shaping spatial heterogeneity in community structure. Functional profiling with FUNGuild revealed that local fungal communities encompassed pathogenic, parasitic, saprotrophic, and symbiotic strategies. Notably, animal pathogens dominated medium-DIN areas, and core functional groups displayed clear spatial patterns along the nutrient gradient. PERMANOVA analysis confirmed significant functional differentiation among the three DIN levels, with DIN accounting for approximately 88.8% of the variation in functional composition and all pairwise comparisons being highly significant—underscoring DIN as the dominant driver of fungal functional organization

Redundancy analysis (RDA) identified depth, salinity, SiO_3_^2−^-Si, pH, COD, Chl *a*, temperature, and DIN as key factors influencing seasonal community variation, with DIN exerting the strongest effect in winter. Together, these findings demonstrate that seasonal dynamics and local nutrient gradients jointly shape fungal community structure and functional composition in the coastal waters of the Leizhou Peninsula.

Collectively, this study provides a comprehensive view of the seasonal, spatial, and functional patterns of marine fungal communities in this understudied region. It highlights the dual influence of temporal and environmental drivers on community assembly and establishes a critical foundation for future research on the ecological roles, adaptive strategies, and conservation of coastal marine fungi.

## Data Availability

The original contributions presented in the study are included in the article/Supplementary Materials; further inquiries can be directed to the corresponding author.

## Supplementary Materials

The following supporting information can be downloaded at: https://www.mdpi.com/article/10.3390/jof12040260/s1, Table S1: Details of the 21 sampling sites in the coastal waters of the Leizhou Peninsula, China; Table S2: Physicochemical variables at the sampling stations in the Leizhou Peninsula, China; Figure S1: Fungal α-diversity indices in relation to DIN gradient (Spearman correlation). (a) Chao1: R = 0.7, *p* = 0.00059; (b) Shannon: R = 0.41, *p* = 0.065; (c) Simpson: R = 0.3, *p* = 0.19.
