# Supplementary material for: Spatiotemporal Dynamics and Co-Occurrence Patterns of Marine Fungal Communities Along Nutrient Gradients in the Leizhou Peninsula, China
Source: J Fungi (Basel). 2026 Apr 3;12(4):260. doi: 10.3390/jof12040260 (PMC13117745; doi:10.3390/jof12040260)
Supplement: Supplementary file 1 [file jof-12-00260-s001.zip › Table S1 Sampling sites.pdf]

Supplementary Table S1. Details of the 21 sampling sites in the coastal waters of the Leizhou Peninsula, China.

Site	Site Name	Site Abbreviation	Longitude	Latitude
S1	Cheban Town (Nuclear Power Plant)	CBZ	109°47'44″	21°28'13"
S2	Anpu Yingzai	APYZ	109°48'54"	21°27'37"
S3	Caotan Town	CTZ	109°45'42"	21°23'23"
S4	Na'ao Bay	NAW	109°50'12″	20°29'54″
S5	Tianchengtai Resort	TCT	109°49'22"	20°33'21"
S6	Xujiagang	XJG	109°52'60″	20°24'44″
S7	Qianshan Town	QSZ	110°33'24″	20°22'26″
S8	Wailuo	WL	110°27'32"	20°34'37"
S9	Xinliao	XL	110°28'22"	20°35'00″
S10	Dongli Town	DLZ	110°20'18"	20°44'30"
S11	Dongnan Port	DNMT	110°30'40″	20°55'17"
S12	Naozhou Island	NZD	110°33'00″	20°53'42"
S13	Longhaitian	LHT	110°32'21"	21°00'26″
S14	Baogang (Steel Industrial Zone)	BG	110°32'12"	21°44'27"
S15	Nansan Island	NSD	110°25'39"	21°10'50″
S16	Techeng Island	TCD	110°25'13"	21°90'48"
S17	Yugang Park	YG	110°25'26"	21°13'70"
S18	Jinsha Bay	JSW	110°23'40″	21°16'00″
S19	Guandu	GD	110°24'50″	21°20'27"
S20	Qiantang Town	QTZ	110°36'12"	21°23'56"
S21	Dinglong Bay	DLW	110°23'40"	21°23'57"
