# Supplementary figures and images for: Spatiotemporal Dynamics and Co-Occurrence Patterns of Marine Fungal Communities Along Nutrient Gradients in the Leizhou Peninsula, China
Source: J Fungi (Basel). 2026 Apr 3;12(4):260. doi: 10.3390/jof12040260 (PMC13117745; doi:10.3390/jof12040260)

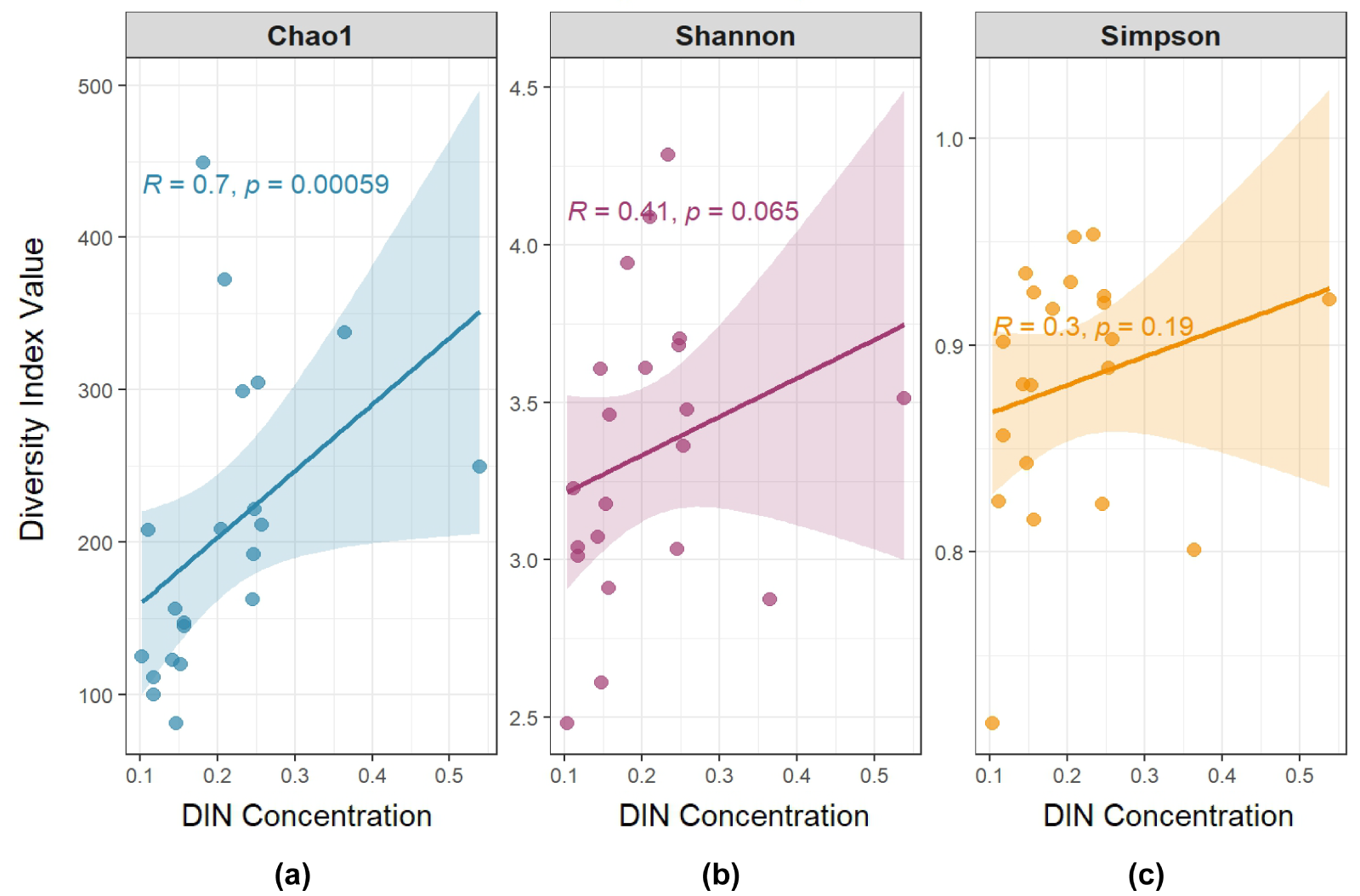

Supplement: Supplementary file 1 [file jof-12-00260-s001.zip › Figure S1 Fungal α-diversity indices in relation to DIN gradient (Spearman correlation).tif]
